# dbAPIS: a database of anti-prokaryotic immune system genes

**DOI:** 10.1093/nar/gkad932

**Published:** 2023-10-27

**Authors:** Yuchen Yan, Jinfang Zheng, Xinpeng Zhang, Yanbin Yin

**Affiliations:** Nebraska Food for Health Center, Department of Food Science and Technology, University of Nebraska - Lincoln, Lincoln, NE 68588, USA; Zhejiang Lab, Hangzhou, Zhejiang 311121, China; Nebraska Food for Health Center, Department of Food Science and Technology, University of Nebraska - Lincoln, Lincoln, NE 68588, USA; Nebraska Food for Health Center, Department of Food Science and Technology, University of Nebraska - Lincoln, Lincoln, NE 68588, USA

## Abstract

Anti-prokaryotic immune system (APIS) proteins, typically encoded by phages, prophages, and plasmids, inhibit prokaryotic immune systems (e.g. restriction modification, toxin-antitoxin, CRISPR-Cas). A growing number of APIS genes have been characterized and dispersed in the literature. Here we developed **dbAPIS** (https://bcb.unl.edu/dbAPIS), as the first literature curated data repository for experimentally verified APIS genes and their associated protein families. The key features of dbAPIS include: (i) experimentally verified APIS genes with their protein sequences, functional annotation, PDB or AlphaFold predicted structures, genomic context, sequence and structural homologs from different microbiome/virome databases; (ii) classification of APIS proteins into sequence-based families and construction of hidden Markov models (HMMs); (iii) user-friendly web interface for data browsing by the inhibited immune system types or by the hosts, and functions for searching and batch downloading of pre-computed data; (iv) Inclusion of all types of APIS proteins (except for anti-CRISPRs) that inhibit a variety of prokaryotic defense systems (e.g. RM, TA, CBASS, Thoeris, Gabija). The current release of dbAPIS contains 41 verified APIS proteins and ∼4400 sequence homologs of 92 families and 38 clans. dbAPIS will facilitate the discovery of novel anti-defense genes and genomic islands in phages, by providing a user-friendly data repository and a web resource for an easy homology search against known APIS proteins.

## Graphical Abstract

**Graphical Abstract:**
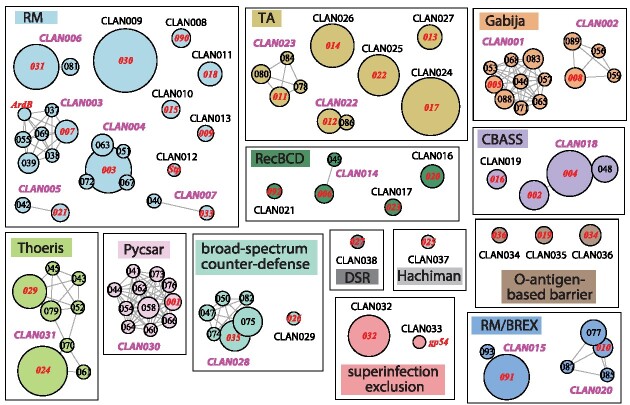

## Introduction

Prokaryotes (bacteria and archaea) are constantly attacked by various mobile genetic elements (MGEs) such as viruses (1), which have an estimated global population >10^31^ (2). Every second, ∼10^25^ phage (bacterial virus) infections are happening globally (3). Prokaryotes and their viruses are under endless arms race for billions of years (4). To avoid infections, prokaryotes have evolved an arsenal of defense mechanisms, also known as the prokaryotic ‘immune systems’ (5). These include the long-known restriction-modification (RM) systems, CRISPR-Cas systems, and toxin-antitoxin (TA) systems, and many more newly discovered ones (3,6,7). Some of these have become very powerful biotechnologies: e.g. CRISPR-Cas9 for genome editing and restriction enzymes for molecular cloning. To overcome these prokaryotic defense systems, MGEs including viruses have evolved anti-defense strategies (8), among which phage encoded anti-CRISPR proteins inhibit CRISPR-Cas systems of their hosts (9), and anti-RM proteins are inhibitors of host's RM systems (10). Identifying new prokaryotic immune systems (PIS) and new anti-PIS (APIS) genes in MGEs has a major significance in the development of new biotechnological tools and the improved understanding of phage-host interactions (6).

Since 2018, the total number of characterized defense systems in prokaryotes has rapidly grown from less than a few to over 100 and will undoubtedly continue to increase in the coming years (7,11). This success is largely due to the application of bioinformatics genome mining of ‘defense islands’ (defense gene clusters) in bacterial genomes followed by highly efficient experimental validations (12,13). Two bioinformatics tools, PADLOC (14) and DefenseFinder (15), become available since 2021 to categorize experimentally characterized PIS protein families, create family hidden Markov models, and build softwares to automatically mine new genomes for these prokaryotic immune systems.

Compared to PIS genes, the study of anti-PIS (or APIS) genes in MGEs is significantly falling behind (16), except for anti-CRISPRs (17), which have over 100 genes experimentally verified (18) (https://tinyurl.com/anti-CRISPR). However, the characterization of new APIS genes inhibiting other prokaryotic immune systems has started to grow remarkably in the literature since 2022 with the notion of ‘anti-defense islands’. Anti-defense islands contain multiple types of APIS genes (e.g. anti-CRISPRs, anti-RMs, and anti-TAs) clustered in genomes of phages and other MGEs (19–22). Currently, there are no online databases to collect APIS genes from biochemical literature (e.g. (19–26)) and no bioinformatics tools for the rapid genome annotation for APIS homologs in MGEs.

We have developed a few bioinformatics tools for anti-CRISPR research (27–31) since 2019. However, no bioinformatics resources exist for other classes of APIS genes. To fill this research gap, we developed dbAPIS (https://bcb.unl.edu/dbAPIS), as the first online database with literature curated data for experimentally verified APIS genes and their associated protein families. Anti-CRISPR proteins are excluded in dbAPIS due to their extensive coverage in existing databases such as anti-CRISPRdb (32).

## Methods

### Literature curation

From a list of most recently published research and review papers/preprints (6,16,19–26) and earlier original papers cited therein, 41 experimentally verified APIS genes were manually extracted. Associated metadata were also curated from these papers and collected from various sources, such as the inhibited prokaryotic immune systems, the APIS protein sequences, functional description, source phages or other MGEs, host taxonomy, PDB structures, sequence and structural homologs, genomic context, etc.

**Figure 1.**
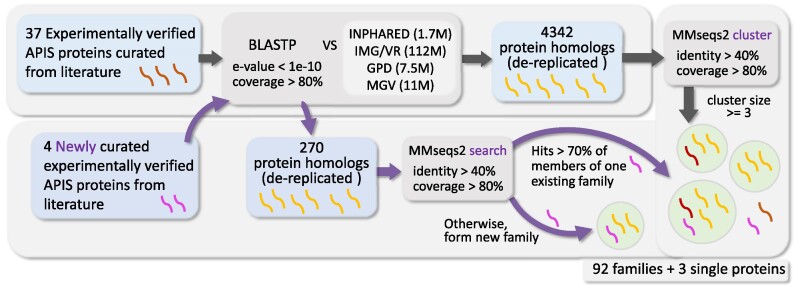
Workflow to create APIS protein families. The top panel shows the creation of the original set of APIS protein families by sequence similarity-based database search and homologous sequence clustering. The bottom panel demonstrates the addition of new APIS families after comparing with existing families. Only sequence clusters with ≥3 sequences form families.

The 41 APIS genes are likely incomplete and represent the result of our literature curation as of June 2023. New APIS genes are continuously being characterized and published. We have designed a computational workflow to routinely incorporate newly curated APIS genes into our existing APIS protein family classification without changing the family names that are already in place. To demonstrate the procedure of creating the original batch of APIS protein family classification and later creating new families, we split the literature-curated 41 APIS proteins (referred to as **seed proteins** hereafter) into two groups: 37 for creating the first batch of families (top panel of Figure 1), and 4 for adding new families (bottom panel of Figure 1).

### Create APIS protein families

37 APIS seed proteins were used to create the first batch of APIS protein families by searching against ∼135 million viral protein sequences from four viral genome databases with BLASTP (33) *E*-value <1e-10 and coverage >80% (top panel of Figure 1). These databases include the INfrastructure for a PHAge REference Database (INPHARED) (34) with all GenBank phage isolate genomes, and three large databases of metagenome assembled (pro)phage genomes: Metagenomic Gut Virus (MGV) (35), IMG/VR (36) and Gut Phage Database (GPD) (37). The identified homologous sequences were processed to remove identical sequences, and then the 4342 de-replicated sequences were combined with all the 37 seed proteins. The final protein sequence set were subject to a sequence clustering using MMseqs2 (38). Using a threshold of within-cluster sequences >40% sequence identity and >80% alignment coverage to form sequence clusters, 89 APIS protein families were created, each with at least three protein members. Among these families, 35 contained seed proteins and the rest did not. Two seed proteins did not have any sequence homologs (Stp, NP_049878.1) or had only one homolog (ArdB, AAB36887.1) in the databases. Therefore, in total 89 protein families (APIS001-089) plus the two seed proteins (Stp and ArdB) formed the first batch of dbAPIS protein families.

### Add new APIS protein families

To demonstrate how to update dbAPIS in the future, we used 4 APIS seed proteins as an example to show the addition of newly curated APIS proteins, the creation of new protein families, and even the deletion of existing families that were wrongly included due to incorrect curation in the past. The 4 seed proteins were first searched against the four viral genome databases to gather homologs (bottom panel of Figure 1) with *E*-value <1e-10 and coverage >80%. Then the 4 seed proteins along with their 270 de-replicated homologs were compared to protein sequences in the existing 89 APIS families and the two single seeds (Stp and ArdB) using MMseqs2 (>40% sequence identity and >80% alignment coverage). If any new seeds and their homologs have hits with >70% members of one existing family, then the proteins are assigned to the family. The remaining protein sequences are used to create new APIS protein families with the MMseqs2 clustering algorithm (within-cluster >40% sequence identity and >80% alignment coverage). As a result of this update, 4 new APIS protein families were created (APIS090-093), 3 of which contained 3 of the new seeds. The fourth seed protein (gp54, YP_001469287.1) did not find any sequence homologs in the databases, and was included in dbAPIS as single seeds like Stp and ArdB. Lastly, one existing family APIS028 (and the associated seed protein) was deleted due to insufficient evidence for the seed protein to be considered as a verified APIS (APIS028 as a void number now). Therefore, the current dbAPIS release contains 92 (89 + 4 – 1) APIS families (38 contains seed proteins) plus 3 single seeds (Stp, ArdB, gp54). It should be noted that the current release of dbAPIS focuses on the experimentally verified APIS genes and their close homologs from four virome/phage databases. It is likely that the three single seeds may have close homologs in other databases such as the RefSeq prokaryotic genomes, prophages, and plasmids. Homologs in these databases will be included in our next release of dbAPIS.

### Build APIS protein family HMMs and select representative proteins

The 92 APIS families contain at least 3 sequences in each family. For each family, sequences were aligned by MAFFT v7.429 (39) and a hidden Markov model (HMM) was built by HMMER v3.3 (40). For the 38 families with seed proteins, the seed protein was selected as the representative protein of the family. For the 54 families without seed proteins, the longest protein from INPHARED was selected as the representative protein. If no proteins are from INPHARED, the representative protein was the one auto-selected by MMseqs2. No HMMs were built for the three single seeds (Stp, gp54, ArdB) as they do not currently form families.

### Group APIS protein families into clans

To find distant homology between the 92 APIS families, HMMs were subject to all-vs-all comparisons using HHsearch v3.3.0 (41) filtered with *E*-value <1e-5. Each clan (a rank higher than family, following the Pfam's protein classification system) contains at least one APIS family with seed proteins and other families without seed proteins in the clan can be inferred with a potential function. For example, four APIS families without seed proteins (063, 067, 072, 051) are predicted to inhibit RM system, as they belong to the same clan as family 003, which contains a seed protein (ArdA, AAB36891.1).

### Gather functional annotation

Member proteins of each APIS family were annotated with Pfam (42) and Prokaryotic Virus Remote Homologous Groups (PHROGs) (43). 3D structures of all member proteins were predicted with AlphaFold2 (44). To find structural homologs of each family, Foldseek (45) was run to search the 3D structure of each representative protein against the AlphaFoldDB (predicted structures of UniProt proteins) and the ESM Metagenomic Atlas (predicted structures of microbiome proteins in MGnify) (46). For each representative protein, its genomic context information (five upstream and five downstream genes in the genome) was extracted.

## Database content

### APIS seed proteins and host immune systems

**Figure 2.**
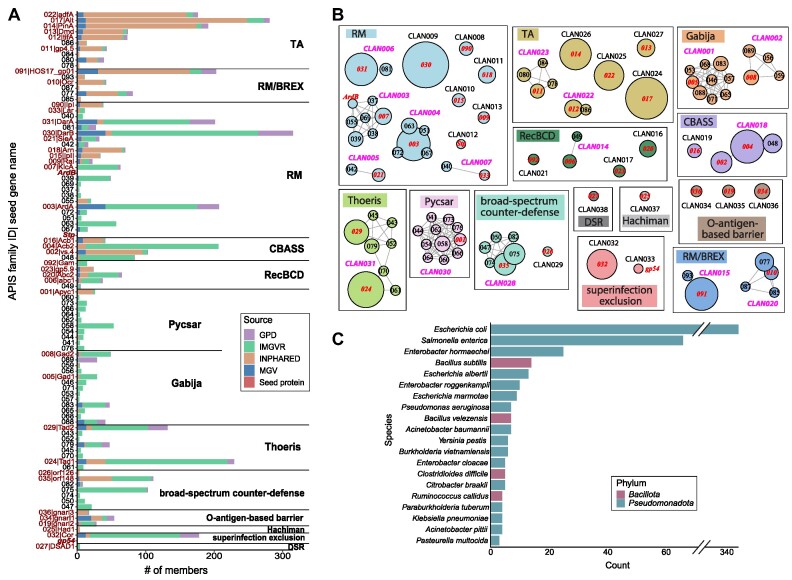
dbAPIS data content and statistics. (**A**) The distribution of APIS family members (seeds + homologs). The family ID (y axis label) is colored in red and indicated with the seed gene name (e.g. 022|adfA) if the family contains seed proteins. The complete member protein list is in Supplementary Table S1. (**B**) Network diagrams to show APIS families/seeds forming clans based on hhsearch homology search. The families are grouped into clans, and clans are grouped by their inhibited defense systems. Each circle represents a family or a single seed (ArdB, Stp and gp54). Each circle is labeled with an APIS family ID and the circle size is proportional to the number of family members. The edge length represents the hhsearch identity. The homology between single seeds (ArdB, Stp and gp54) and families are obtained using hhblits. The circle label is colored in red if the family contains seed proteins. The clans are colored by inhibited defense systems. There are 16 clans with ≥2 families, and 22 clans with a single family or a single seed protein. (**C**) The top 20 host species distribution of APIS seeds and homologs.

The current release of dbAPIS contains in total 41 experimentally verified seed APIS proteins (red fonts in Figure 2A). Some of the 41 seed proteins were characterized decades ago and have conserved Pfam domains named after them, e.g. ArdA (PF07275), DarA (PF18788), Dmd (PF17587), Gam (PF06064), Ral (PF11058), Ocr (PF08684) (Supplementary Table S1). However, 22 of the 41 seed proteins have no conserved domain match in Pfam, e.g. the most recently characterized Acb1 (23), Acb2 (26), Gad1 (47), Gad2 (47), Tad1 (25), Tad2 (47).

From the curated literature, these 41 proteins are classified into 13 groups according to their inhibited host defense systems (Figure 2A): Restriction-Modification (RM, 14 proteins), Toxin-Antitoxin (TA, 6), Recombinational Repair of Double-Stranded DNA Breaks (RecBCD, 4), Cyclic oligonucleotide-Based Antiphage Signaling System (CBASS, 3), O-antigen-based barrier (3), Gabija (2), Bacteriophage Exclusion (BREX, 2), Thoeris (2), broad-spectrum counter-defense (2), superinfection exclusion (2), Pyrimidine cyclase system for antiphage resistance (Pycsar, 1), Hachiman (1), defence-associated sirtuin (DSR, 1). Two seed proteins (Ocr|APIS010 and Gam|APIS092) can inhibit both RM and BREX systems and thus belong to two groups.

### APIS protein families and clans

The 41 APIS seed proteins and their homologs (in total 4428 sequences, Supplementary Table S1) are classified into 92 families plus 3 single seeds (Figure 2A). A family contains protein sequences with sequence identity >40%, while a clan (or superfamily) contains a collection of families that share more distant sequence similarities. Out of the 92 families, 51 have conserved Pfam domains, and 38 contain seed proteins; 72 (18 with seeds) of the 92 families are further grouped into 16 clans (pink color in Figure 2B). The other 20 families (each has a seed) form 20 single-family clans. Of the three single seeds, ArdB belongs to CLAN003 (KlcA), while Stp and gp54 each defines their own clan. Therefore, in total 38 (16 + 20 + 2) clans are formed each containing a seed protein (red color in Figure 2B), and thus are annotated with a function in terms of their inhibited host defense systems according to the contained seed proteins. The RM system has the most APIS families (24) and clans (11), followed by the TA system (10 families and 6 clans). Note anti-CRISPRs are not included in dbAPIS, and over 100 anti-CRISPR families have been characterized already (18) (https://tinyurl.com/anti-CRISPR).

The APIS family sizes vary significantly (Figure 2A), with the number of members ranging from three to over 300 (e.g. APIS030|DarB). Most APIS homologs are from IMG/VR, particularly in families lacking seed proteins (e.g. most families in Pycsar, RM, Gabija). This prevalence suggests that the IMG/VR database could potentially harbor a broader diversity of undiscovered APIS proteins. INPHARED, which contains only isolate phage genomes, also accounts for a large portion of some families, and is most abundant in TA inhibiting APIS families. The two gut virome databases GPD and MGV are widely distributed in families with seed proteins, but seem to be absent in Pycsar inhibiting APIS families.

Prokaryotic host information was extracted from literature and the four source virome databases. The top 20 host species with the most APIS proteins (Figure 2C) belong to the Pseudomonadota and Bacillota phyla. *Escherichia coli* is the host species of 345 APIS proteins, much more than other species. This agrees with the fact that 21 of the 41 APIS seed proteins were characterized from phages of *Escherichia coli* (10 from T4 phage). It does not mean *Escherichia coli* phages have more APIS genes, but only reflects the biased experimental phage system used for APIS discovery. However, the APIS homologs do exhibit a broad host taxonomic distribution, indicating the broad existence of APIS in phages infecting an extensive biological diversity of prokaryotic hosts. This is consistent with the wide distribution of various prokaryotic immune systems against phages.

## Web design

The dbAPIS website is powered by MySQL + PHP + JavaScript + Apache + HTML. It has the following major components:

**Figure 3.**
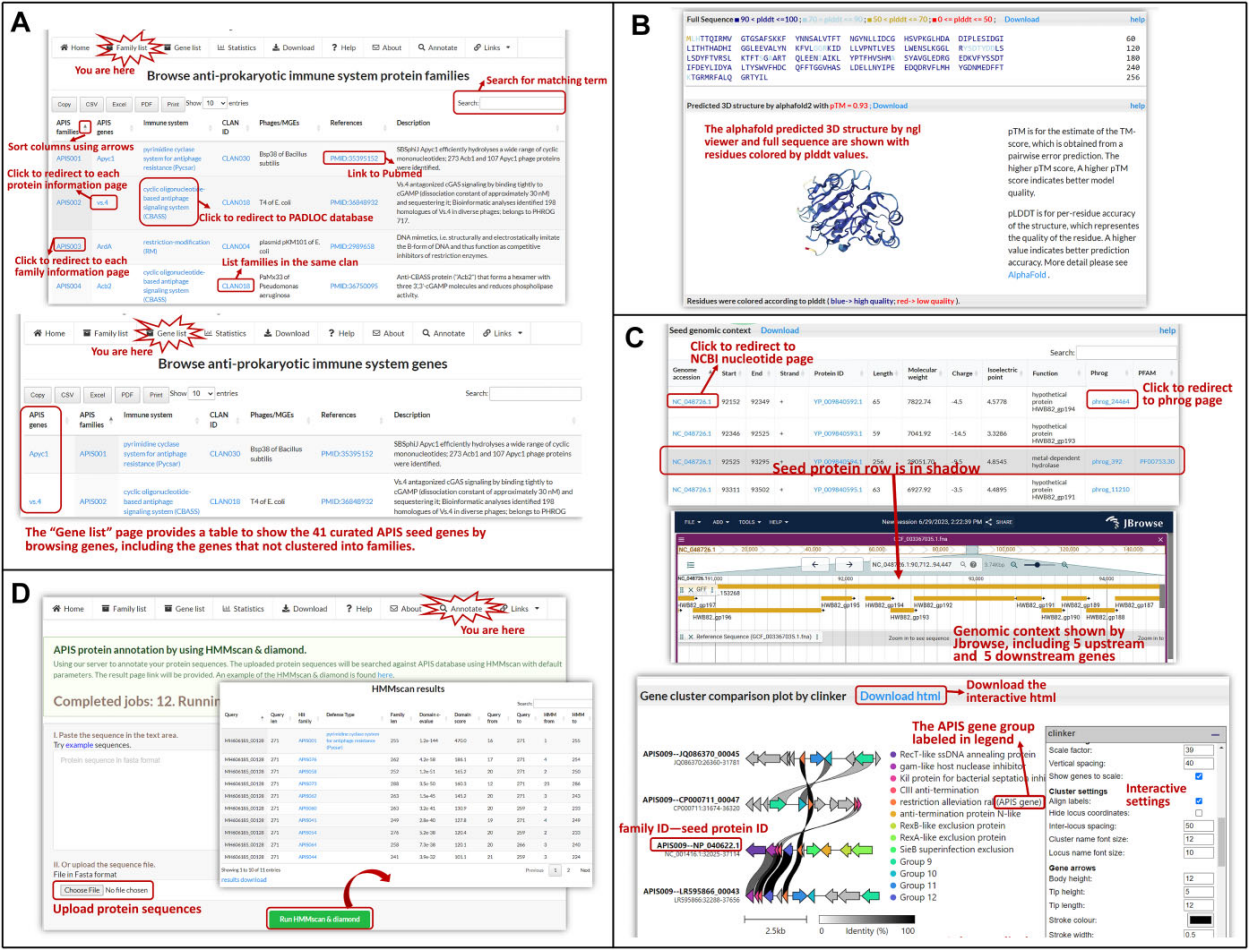
Screenshots of dbAPIS website. (**A**) The family and gene lists for browsing. (**B**) An example seed protein 3D structure page (*https://bcb.unl.edu/dbAPIS/gene_page.php?prot_id=YP_009840594.1*). (**C**) Genomic context information of member proteins (*https://bcb.unl.edu/dbAPIS/anti_defense_family.php?family_id=APIS001#Seed_genomic_context*) and the gene cluster comparison plot using Clinker (*https://bcb.unl.edu/dbAPIS/anti_defense_family.php?family_id=APIS009#Family_members*). The embedded Clinker viewer is interactive, and users can refer to the control menu to adjust the plot. (**D**) HMMscan & DIAMOND annotation page for searching APIS sequence homologs.

### Family and gene lists (Figure 3A)

The ‘Family list’ in the navigation bar provides information of the 92 APIS family IDs, clan IDs, gene names, inhibited immune systems, and PubMed IDs as well as gene descriptions. The ‘Gene list’ page provides a table to show the 41 curated APIS seed genes. Clicking on the ‘APIS families’ column in the two pages will open the family page with various information:

- *Seed protein information*: Metadata about the seed protein of the family are shown, such as protein accession, gene location, phage property, host taxonomy. Links are provided to direct users to external databases such as GenBank, Taxonomy, Pfam, PHROGs, PADLOC databases.
- *Seed protein genomic context:*Gene information of the seed protein along with its five upstream and five downstream genes is tabulated, including location, strand, sequence length, molecular weight, charge, isoelectric point, PHROGs and Pfam annotation, as well as genomic context visualization using JBrowser.
- *Seed protein 3D structure*: If the protein has PDB structure information, then the PDB structure and full sequence are shown; if not, the AlphaFold predicted 3D structure by ngl viewer and full sequence are shown with residues colored by plddt values (Figure 3B).
- *Structure homologs:*The links to Foldseek results in HTML and tabular format for protein structure homologs from searching against AlphaFoldDB and ESM Metagenomic Atlas database are provided, respectively.
- *Family information:*The protein sequence in FASTA format, multiple sequence alignment and HMM model of the family are provided for download. The host taxonomy and sequence length distribution are represented with plots.
- *Clan information and family members:*The clan table lists families that share sequence similarity according to *hhsearch*.
- *Family members:*For members in each family, we provide the BLASTP results of the representative protein against each member, along with the corresponding host and source database. Users can access the individual protein page that contains AlphaFold predicted structures for each protein homologs. We also gathered the genomic context information of member proteins, and generated the gene cluster (five upstream and five downstream genes of the member protein) comparison plot using Clinker (48) (Figure 3C). Such plot is interactive, customizable, and very valuable for users to visualize the gene neighborhood conservation among the member proteins of the family.

### Annotation page

With the APIS family HMMs and APIS protein sequences, we allow users to submit their own protein sequences to our server for an automated search of APIS sequence homologs (Figure 3D). The query sequences will be taken for HMMscan and DIAMOND (49) runs on our server. The results will be returned on the web as tables, and can also be downloaded as text files. This function will help users quickly identify the homologs of APIS proteins in the submitted sequences.

### Download page

We provide the HMMs and the FASTA sequences of all APIS proteins (seeds and homologs). Users can download them in batch and run the APIS gene annotation on their own computers.

## Conclusions

dbAPIS provides the first web resource for experimentally verified anti-prokaryotic immune system (APIS) genes in phages and other mobile elements along with abundant metadata. We started with 41 seed proteins and included ∼4400 sequence homologs (Supplementary Table S1). It will be a useful bioinformatics tool for the genome-based discovery of novel anti-defense systems in phages. The website features various manually curated data from literature and pre-computed data on the genomic context, structure and homology information of APIS proteins. These features will help a broad community of microbial researchers to study (i) phage-host interactions, and (ii) bacterial immunity. dbAPIS will contribute to the development of new biotechnologies for: (i) phage therapy to combat antibiotic resistance of infection diseases, and (ii) precision modulation of existing or new molecular biology tools (examples: RM for molecular cloning and CRISPR-Cas for genome editing). Therefore, as the first of its kind, dbAPIS will be a novel and important manually curated web resource for microbial biologists to study the anti-defense mechanisms of phages.

We will update dbAPIS at least once a year to include newly curated APIS genes from literature and create new APIS protein families/clans in each update. The current database focuses on the verified APIS genes, and only included their close homologs from four virome/phage databases. Future development of dbAPIS will expand its scope to include homologs from prokaryotic genomes, prophages, and plasmids, as well as anti-CRISPR proteins and their homologs. We will also develop new bioinformatics tools for the automated genome mining of anti-defense islands using the APIS family HMMs to facilitate the rapid discovery of new APIS genes from the ever increasing virome data.

## Supplementary Material

gkad932_Supplemental_Files

## Data Availability

All data are available at https://bcb.unl.edu/dbAPIS.
