# Supplementary material for: dbAPIS: a database of anti-prokaryotic immune system genes
Source: Nucleic Acids Res. 2023 Oct 27;52(D1):D419–25. doi: 10.1093/nar/gkad932 (PMC10767833; doi:10.1093/nar/gkad932)
Supplement: gkad932_Supplemental_Files [file gkad932_supplemental_files.zip › SUPPLEMENTARY TABLES.docx]

**SUPPLEMENTARY TABLES**

**Table S1:** Classification of 4,428 APIS protein sequences in dbAPIS (clan, family, inhibited immune systems, Pfam, PHROG, host)
